# p53/TAp63 and AKT Regulate Mammalian Target of Rapamycin Complex 1 (mTORC1) Signaling through Two Independent Parallel Pathways in the Presence of DNA Damage*

**DOI:** 10.1074/jbc.M113.530303

**Published:** 2013-12-23

**Authors:** Maren Cam, Hemant K. Bid, Linlin Xiao, Gerard P. Zambetti, Peter J. Houghton, Hakan Cam

**Affiliations:** From the ‡ Center for Childhood Cancer and Blood Diseases, Nationwide Children's Hospital, Columbus, Ohio 43205,; the § Department of Biochemistry, St. Jude Children's Research Hospital, Memphis, Tennessee 38105, and; the ¶ Department of Pediatrics, College of Medicine, Ohio State University, Columbus, Ohio 43205

**Keywords:** Akt, DNA Damage Response, mTOR, mTOR Complex (mTORC), p53, p63, 4EBP1, REDD1, S6K1, Sestrin

## Abstract

Under conditions of DNA damage, the mammalian target of rapamycin complex 1 (mTORC1) is inhibited, preventing cell cycle progression and conserving cellular energy by suppressing translation. We show that suppression of mTORC1 signaling to 4E-BP1 requires the coordinated activity of two tumor suppressors, p53 and p63. In contrast, suppression of S6K1 and ribosomal protein S6 phosphorylation by DNA damage is Akt-dependent. We find that loss of either p53, required for the induction of Sestrin 1/2, or p63, required for the induction of REDD1 and activation of the tuberous sclerosis complex, prevents the DNA damage-induced suppression of mTORC1 signaling. These data indicate that the negative regulation of cap-dependent translation by mTORC1 inhibition subsequent to DNA damage is abrogated in most human cancers.

## Teaser

**Background:** mTORC1 integrates cellular inputs and is often deregulated in cancer.

**Results:** In response to DNA damage, p53/TAp63 and AKT regulate mTORC1 through two independent parallel pathways.

**Conclusion:** DNA damage activates Akt, resulting in inhibition of S6K1, whereas Sestrin1/2 downstream of p53 and REDD1 downstream of p63 coordinate to suppress 4E-BP1.

**Significance:** mTORC1-dependent 4EBP1 inhibition by DNA damage is abrogated in most human cancers.

## Introduction

The mammalian target of rapamycin (mTOR)^2^ is a major controller of growth and is often deregulated in cancer and diabetes (1–3). mTOR belongs to the family of PI3K-related kinases, is highly conserved from yeast to human, and exists in two distinct complexes termed complex 1 (mTORC1) and complex 2 (mTORC2). mTORC1 has been studied most extensively and comprises mTOR, Raptor, and mLST8. Multiple pathways, including mitogenic growth factors, hormones such as insulin, cellular energy levels, nutrients (amino acids and glucose), and stress conditions tightly control the activation status of mTORC1 (4).

In the presence of the appropriate growth signals, activated mTORC1 controls growth (increase in cell mass) and proliferation (increase in cell number) by modulating mRNA translation through phosphorylation of the eukaryotic translation initiation factor 4E (eIF4E)-binding proteins (4E-BP1, 2, and 3) and the ribosomal protein S6 kinases (S6K1 and 2). 4E-BPs regulate the translation of a subset of mRNAs by competing with eIF4G for binding to eIF4E, thus preventing the assembly of the eIF4F complex. The S6Ks control the phosphorylation status of a number of translational components, including small ribosomal protein S6 (rpS6) and eIF4B, which is required for efficient recruitment of ribosomes to mRNA (5). However, under stress conditions, mTORC1 signaling is suppressed (6–9), allowing for energy conservation, recycling of cellular components (autophagy), and survival until conditions normalize. Thus, by integrating intra- and extracellular signals, the mTORC1 complex plays a crucial role in maintaining cellular homeostasis under conditions of normal proliferation and stress. Interestingly, in many cancers, mTORC1 signaling is enhanced, even under conditions where such signaling should be suppressed. It has been shown previously that, in response to genotoxic stress, the tumor suppressor protein p53 is activated and inhibits mTORC1 signaling by inducing the transcription of the SESTRIN1/2 genes (10). Subsequently, induced SESTRIN1/2 activates AMPK by an unknown mechanism and results in the suppression of mTORC1 signaling. Moreover, it has been reported that mTORC1 activity could also be inhibited through p53-dependent but SESTRIN1/2-independent up-regulation of known mTORC1 negative regulators (11, 12). In addition to repressing mTORC1 via transcriptional targets, indirect evidence suggests that p53 causes a rapid decrease in translation initiation, partly by regulating the phosphorylation of S6K and 4E-BP1 (13). Interestingly, the tuberous sclerosis complex (TSC) is suggested not to be required for the inhibition of the mTORC1 pathway under certain cellular stress conditions that activate p53 (14). Moreover, TSC2^+/+^p53^−/−^ MEFs, irradiated and treated with H_2_O_2_ or etoposide, were able to suppress mTORC1 signaling, as determined by monitoring the phosphorylation status of S6K1 (p70S6K) (15). Taken together, these data indicate that the regulation of mTORC1 signaling under conditions of DNA damage is more complex than anticipated and suggests the existence of alternative signaling pathways in the regulation of mTORC1.

To test this conjecture, we explored the upstream regulation of mTORC1 signaling under conditions of drug-induced DNA damage using genetically defined MEFs as well as *in vivo* tumor models. DNA damage-induced activation of 4E-BP1, through dephosphorylation, required the simultaneous induction of both Sestrin1/2 and REDD1 under the control of p53 and p63 tumor suppressor proteins, respectively. In contrast, in p53-deficient cells and tumors, DNA damage-induced hypophosphorylation of ribosomal S6 protein or inactivation of S6Ks was dependent on a novel signaling cascade through which the DNA-PK/Akt axis can still restrict cellular metabolism independently of p53.

## EXPERIMENTAL PROCEDURES

### 

#### 

##### Animal Experiments

CB17SC-F *scid*^−/−^ mice (Taconic Farms, Germantown, NY) were used to propagate subcutaneously implanted tumors. All mice were maintained under barrier conditions, and experiments were conducted using protocols and conditions approved by the institutional animal care and use committee. When tumors reached 200–300 mm^3^, topotecan (2 mg/kg daily for 2 days) or saline, as a control, was administered by intraperitoneal injection. Snap-frozen samples were lysed and analyzed by immunoblotting.

##### Cell Culture and Treatments

All MEFs, as well as the human breast cancer cell lines MCF-7 and MDA-MB-231, were cultured in DMEM (Lonza) supplemented with 10% FBS (Sigma) and penicillin-streptomycin (Invitrogen). The human non-small lung cancer cell lines A549 and H1299, the human prostate cancer cell lines LNCaP and PC3, the human Ewing sarcoma cell lines ES2 and ES4, as well as the human rhabdomyosarcoma cell lines Rh18 and Rh30 were cultured in RPMI 1640 medium (Corning) supplemented with 10% FBS and penicillin-streptomycin. SK-N-SH cells were cultured in Eagle's minimal essential medium (Corning) supplemented with 10% FBS and penicillin-streptomycin. SK-N-BE(2) cells were cultured in EMEM:Ham's F12 medium (1:1) (Corning) supplemented with 10% FBS and penicillin-streptomycin. Normal human dermal fibroblasts (NHDFs) were obtained from the ATCC and cultured in fibroblast basal medium (ATCC, catalog no. PCS-201-030) supplemented with fibroblast growth kit low serum (ATCC, catalog no. PCS-201-041) and penicillin-streptomycin (Invitrogen). Topotecan, etoposide, and cisplatin were purchased from Sigma. AZD8055 and AZD6244 were provided by AstraZeneca. The caspase inhibitor carbobenzoxy-valyl-alanyl-aspartyl-(*O*-methyl)-fluoromethylketone (ZVAD-FMK) was obtained from MBL International. Unless indicated otherwise, all drugs and DNA damage-inducing agents were applied in the presence of FBS. siRNA oligonucleotides targeting human SESTRIN2, REDD1, or non-silencing were purchased from Dharmacon and transfected using the Lipofectamine 2000 transfection reagent (Invitrogen).

##### Immunoblot Analysis, Immunoprecipitation, and S6K1 Kinase Assay

Cells were lysed on ice with lysis buffer (Cell Signaling Technology) supplemented with protease inhibitors (Roche) and 1 mm PMSF (Sigma). For immunoprecipitation, precleared lysates were incubated with either pan-14-3-3 mouse monoclonal antibody (Ab-4, LabVision) or mouse monoclonal AMPK-α (Abcam) for 24 h at 4°C. Immunocomplexes were precipitated using protein G-Sepharose (Amersham Biosciences) and then washed four times with lysis buffer prior to analysis by SDS-PAGE. Immunoblot analyses were probed with the following antibodies. Antibodies to detect p53 of human and mouse origin were purchased from Santa Cruz Biotechnology and BD Biosciences, respectively. Anti-Bak antibody was obtained from Sigma. Antibodies for the detection of β-actin, Bax, DNA-PK, p21, and p63 were purchased from Santa Cruz Biotechnology. Anti-γ-H2AX antibody was bought from Upstate. Anti HA-antibody was obtained from Covance. REDD1 and anti-Sestrin2 antibodies were purchased from ProteinTech Group. AMPK-α, AMPK (p-T172), poly (ADP-ribose) polymerase (PARP), S6, S6 (p-Ser-235/236), 4E-BP1, 4E-BP1 (p-Thr-37/46), 4E-BP1 (p-*Thr-70*), p70S6 kinase (p-Thr-389), cleaved caspase 3, caspase 9, Akt, Akt (p-Ser-473), Akt (p-Thr-308), c-Raf, c-Raf (p-Ser-259), ERK, ERK (p-Thr-202/Tyr-204), MEK1/2, MEK1/2 (p-Ser-217/221), S6K1/2, TSC2, p53 (p-Ser-15), and ATM were detected using Cell Signaling Technology antibodies. For the S6K1 kinase activity assay, precleared lysates were immunoprecipitated with specific anti-S6K1 antibody (Cell Signaling Technology) or control non-immune rabbit immunoglobulin for 24h at 4 °C. *In vitro* kinase assays were performed using a synthetic peptide substrate (AKRRRLSSLRA), and S6K1 kinase activity was measured using an S6 kinase assay kit (Upstate Biotechnology-Millipore Inc.) according to the instructions of the manufacturer. Values were calculated by subtracting nonspecific activity, detected in rabbit IgG immunoprecipitates, from kinase activity detected in anti-S6K1 immunoprecipitates.

##### Plasmid Construction, Mutagenesis, and Luciferase Assay

Full-length human SESTRIN2 cDNA was generated from DNA damage-treated NHDFs and cloned into pCMV-Tag2a (Stratagene) using the following primer pair: atcggatcccatgatcgtggcggactcc (forward) and atcggatcctcaggtcatgtagcgggtgat (reverse). The pCMV-Tag2a-REDD1 construct was generated by subcloning of REDD1 from the pCDNA.3-HA-REDD1 plasmid (provided by Leif W. Ellisen). Mutant constructs of TAp63γ were generated by site-directed mutagenesis (Stratagene). The TAp63γ mutant contains alanine substitutions at Ser-42 or Thr-44. The REDD1 reporter luciferase construct (provided by Leif W. Ellisen) was cotransfected with TAp63α or TAp63γ, and DNA binding mutants of TAp63α or γ isoforms were provided by Dr. Kurt Engeland. A luciferase assay was performed according to the instructions of the manufacturer (Promega). The pcDNA3 Myr-Akt1 plasmid was from William Sellers (Addgene plasmid 9008).

##### Statistical Analysis

All data analysis was performed using GraphPad Prism Version 5. Bar graphs represent mean ± S.D. or S.E. as indicated. Statistical significance was assessed using Student's *t* test (*p* < 0.05).

## RESULTS

### 

#### 

##### Analysis of mTORC1 Signaling in Tumor Cell Lines and Xenograft Models in the Presence of DNA Damage

**FIGURE 1.**
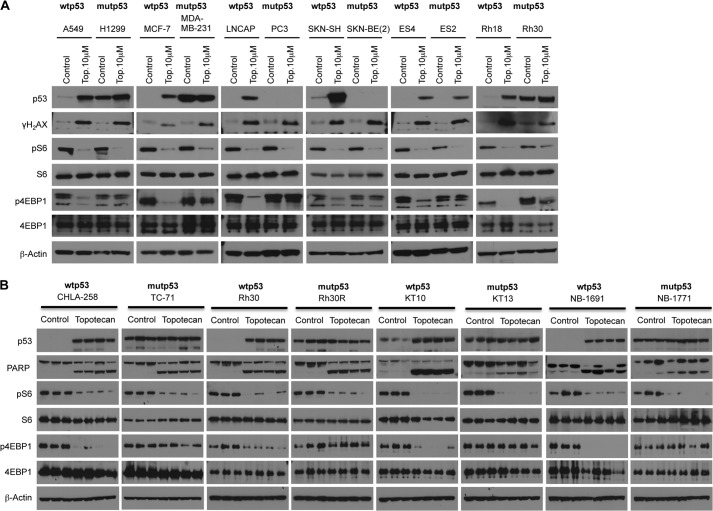
**Analysis of mTORC1 signaling in tumor cell lines and xenografts in the presence of DNA damage.** *A*, tumor cell lines were treated with dimethyl sulfoxide or 10 μm topotecan (*Top.*) for 24 h, and the cell lysates were immunoblotted with the indicated antibodies. The p53 genotype is shown above each line. *mut*, mutant. *B*, mice bearing tumor xenografts derived from Ewing sarcoma (CHLA-258 and TC-71), rhabdomyosarcoma (Rh30 and Rh30R), Wilms tumor (KT-10 and KT-13), neuroblastoma (NB-1691 and NB-1771) were treated with dimethyl sulfoxide or with topotecan (2 mg/kg daily) for 2 days. Snap-frozen samples were lysed and immunoblotted with the indicated antibodies. The p53 genotype is shown above each line. Note that Rh30 cells *in vitro* have the same p53 mutation as in Rh30R xenografts (R273C), whereas the Rh30 xenograft is WT p53.

The relationship between the DNA damage-induced metabolic checkpoint and mTORC1 inhibition has not been studied extensively in human tumor cell lines (7). Thus, we elucidated the effects of drug-induced DNA damage on mTORC1 signaling in various tumor cell lines as well as xenograft tumor models. As shown in Fig. 1*A*, we treated tumor cells from different adult and childhood tumors or xenograft tumors (Fig. 1*B*) representing several pediatric histotypes harboring either functional or non-functional p53 with topotecan. Surprisingly, the treatment of tumor cell lines induced dephosphorylation of rpS6 regardless of the p53 status. However, we found that only p53 wild-type tumor cell lines are able to suppress 4E-BP1 phosphorylation in the presence of DNA damage, indicating that p53 protein is required for the inhibition of 4E-BP1 but not rpS6. It is well established that the tumor microenvironment plays a crucial role in the response to DNA damage. Thus, we next determined the drug-induced DNA damage on mTORC1 signaling *in vivo*. As shown in Fig. 1*B*, similar to treated tumor cell lines, topotecan treatment induced dephosphorylation of rpS6 regardless of the p53 status of the xenografts, whereas 4E-BP1 dephosphorylation occurred only in the presence of functional p53. Taken together, these data suggest that there may be both quantitative and qualitative differences in the translation in response to DNA damage induced by cancer chemotherapeutic agents, dependent on the functional status of p53.

##### Regulation of Ribosomal Protein S6 in Response to DNA Damage Is Akt-dependent

**FIGURE 2.**
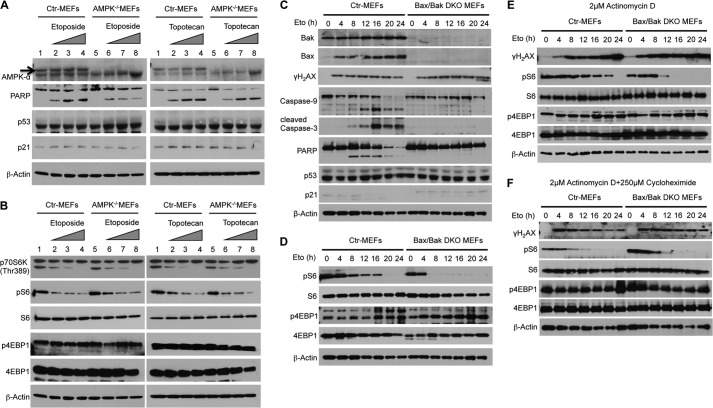
**AMPK and p53 are not required for the inhibition of rpS6 phosphorylation in response to DNA damage.** *A* and *B*, immortalized control (*Ctr*) and AMPKα^−/−^ MEFs were treated with increasing concentrations of etoposide or topotecan for 24 h. Cell extracts were analyzed by Western blot analysis with antibodies as shown. *C*, immortalized control or Bax/Bak DKO MEFs were treated with etoposide (*Eto*) (20 μm) for the indicated times. Cell extracts were analyzed by Western blot analysis with antibodies as shown. DNA damage induces apoptosis only in WT MEFs. *D*, DNA damage induces dephosphorylation of rpS6 protein in both control and Bax/Bak DKO MEFs. *E* and *F*, the inhibition of new transcription or protein synthesis in the presence of DNA damage has no impact on rpS6 regulation. MEFs were treated with 2 μm actinomycin D (*E*) alone or in combination with 250 μm cycloheximide (*F*) for the indicated times. Cell extracts were analyzed by Western blot analysis with antibodies as shown.

Previous reports implied that AMPK might be a key factor for the regulation of mTORC1 in the presence of DNA damage. However, direct evidence demonstrating how AMPK suppresses mTORC1 signaling under classical DNA damage conditions is missing. Surprisingly, regardless of the AMPK and p53 status of the treated MEFs (Fig. 2*A*), DNA damage induced a dose-dependent inactivation of S6K1 (p70S6K) and the subsequent dephosphorylation of rpS6 (Fig. 2*B*). However, in response to DNA damage, both immortalized wild-type and AMPK^−/−^ MEFs were unable to suppress 4E-BP1 phosphorylation, which indicates a possible p53- and/or AMPK-dependent mechanism of mTORC1 regulation (Fig. 2*B*).

We next focused on AMPK- and p53-independent down-regulation of rpS6 phosphorylation in response to DNA damage. Initially, we observed that increased concentrations of DNA damaging agents resulted in increased apoptosis that correlated with the suppression of S6K1 (p70S6K) and the subsequent dephosphorylation of rpS6 (data not shown). This suggested that the level of apoptosis might be the key factor to suppress rpS6 phosphorylation. Thus, we compared SV40-transformed Bax/Bak DKO MEFs, which cannot undergo intrinsic pathway-induced apoptosis, with control MEFs in the presence of etoposide (Fig. 2*C*). In contrast to control MEFs, Bax/Bak DKO MEFs do not activate caspase 3/9 or cleave poly (ADP-ribose) polymerase (PARP). However, the levels of DNA damage appear similar between wild-type and DKO MEFs, as shown by the induction of γH2AX, a marker of DNA strand breaks. As shown in Fig. 2*D*, treatment with etoposide in both MEF strains resulted in the suppression of rpS6 phosphorylation. In addition, the inhibition of apoptosis by ZVAD-FMK showed no difference between treated AMPK^−/−^ and its control MEFs for the suppression of rpS6 (data not shown). Consistent with our initial experiments with AMPK^−/−^ MEFs, both Bax/Bak DKO and control MEFs were also not able to down-regulate 4E-BP1 phosphorylation in the presence of DNA damage (Fig. 2*D*). Our results indicate that p53- and AMPK-independent regulation of rpS6 is not simply dependent on the consequences of apoptosis, and they also indicate that new transcription, protein synthesis, or phosphorylation events might be needed to regulate rpS6. Accordingly, we treated Bax/Bak DKO and control MEFs with actinomycin D alone (Fig. 2*E*) or in combination with cycloheximide (Fig. 2*F*). As shown in Fig. 2, *E* and *F*, neither inhibition of transcription nor translation had an effect on rpS6 phosphorylation, suggesting the existence of a signaling cascade independently of mTORC1 that might control phosphorylation of rpS6 in response to DNA damage.

**FIGURE 3.**
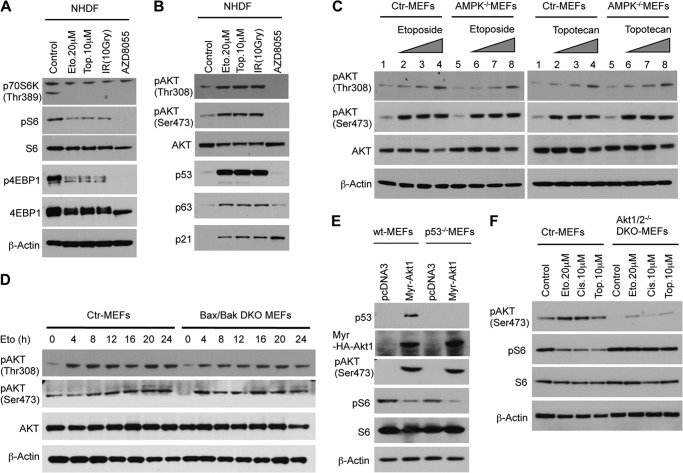
**DNA damage significantly increases Akt phosphorylation.** *A*, NHDFs were treated for 48 h with the indicated DNA-damaging agents (*Eto*, etoposide; *Top*, topotecan; *IR*, ionizing radiation) or with AZD8055, a specific mTOR inhibitor. *B*, DNA damage induces phosphorylation of Akt and activates p53 and p63 and their target protein p21. *C*, control (*Ctrl*) and AMPKα^−/−^ MEFs were treated with increasing concentrations of etoposide or topotecan for 24 h. *D*, control and Bax/BakDKO MEFs were treated with etoposide (20 μm) for the indicated times. Cell extracts were analyzed by Western blot analysis with antibodies as shown. *E*, introduction of myr-Akt, a constitutively active form of Akt, suppresses rpS6 phosphorylation in WT or p53^−/−^ MEFs. 48 h after electroporation, cell extracts were analyzed by Western blot analysis with antibodies as shown. *F*, DNA damage dependent regulation of rpS6 phosphorylation is abolished in Akt1/2^−/−^ MEFs. Immortalized MEFs were treated with indicated DNA damaging agents for 24 h. *Cis*, cisplatin.

The inactive Akt-protein kinase B (PKB) complex can be rapidly activated by growth factors via the PI3K-PDK1 and mTORC2 pathways, which leads to phosphorylation of Akt-PKB at Thr-308 and Ser-473 (16). Activated Akt mediates survival by transcription and posttranslational modification of genes and proteins involved in apoptosis. However, it has been reported previously that Akt is also phosphorylated at both residues in response to DNA damage (17–19). To analyze Akt phosphorylation, we treated NHDFs with different DNA-damaging stimuli, which completely suppressed mTORC1 signaling (Fig. 3*A*). Surprisingly, DNA damage significantly increased Akt phosphorylation in NHDFs (Fig. 3*B*), AMPK^−/−^_,_ Bax/Bak DKO MEFs (Fig. 3, *C* and *D*), in various tumor cell lines, as well as in xenograft tumor models (data not shown). These observations led us to investigate the ability of myr-Akt, a constitutively active form of Akt, to control rpS6 phosphorylation. Thus, we transfected WT MEFs as well as p53^−/−^ MEFs with myr-Akt or a control plasmid. Expression of myr-Akt, in MEFs, regardless of their p53 status, induced dephosphorylation of rpS6 (Fig. 3*E*), which indicates that the activation of Akt might regulate rpS6 phosphorylation. Furthermore, in contrast to immortalized control MEFs, immortalized Akt1/2 DKO MEFs treated with DNA-damaging agents were not able to suppress rpS6 phosphorylation, supporting our conjecture that activation of the Akt pathway plays a key role in the regulation of rpS6 phosphorylation in response to DNA damage (Fig. 3*F*).

**FIGURE 4.**
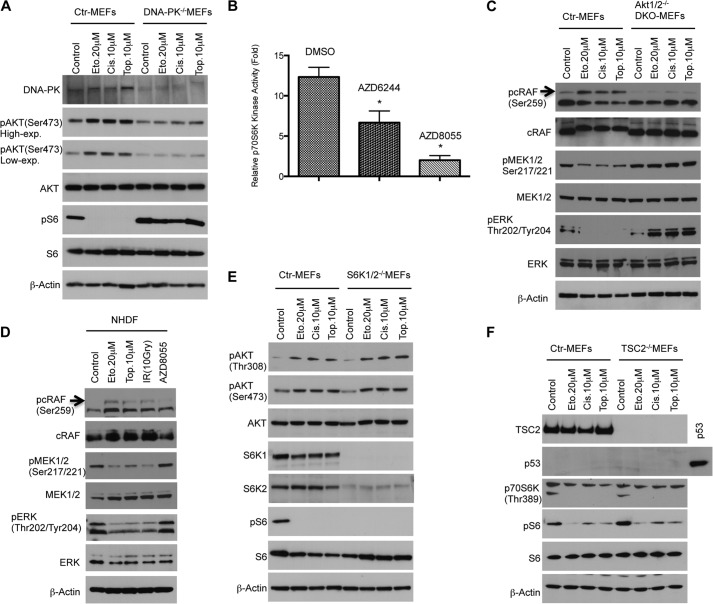
**Regulation of ribosomal protein S6 in the presence of DNA damage is mediated by the DNA-PK/Akt/c-Raf/MEK/MAPK/S6K1 signaling cascade.** *A*, DNA-PK is required for activation of Akt and inhibition of rpS6 under DNA damage conditions. Control (*Ctr*) or DNA-PK MEFs were treated with indicated DNA-damaging agents for 24 h. Cell extracts were analyzed by Western blot analysis with antibodies as shown. *Eto*, etoposide; *Cis*, cisplatin; *Top*, topotecan. *B*, inhibition of MEK signaling by a specific MEK inhibitor, AZD6244, reduces S6K1 (p70S6K) kinase activity. After NHDFs were treated with the indicated drugs or dimethyl sulfoxide (*DMSO*) as a control for 24 h, 10 μg of protein for each condition was immunoprecipitated with specific anti-S6K1 antibody or control non-immune rabbit immunoglobulin. *In vitro* kinase assays were performed as described under “Experimental Procedures.” *Error bars* show the mean ± S.D. for duplicate plates in a representative experiment. *, *p* < 0.05. *C*, negative regulation of ERK1/2 is mediated by Akt-induced phosphorylation of c-Raf under conditions that damage DNA. Control and Akt1/2^−/−^ DKO MEFs were treated with the indicated DNA-damaging agents, and cell extracts were analyzed by Western blot analysis with antibodies as shown. *D*, NHDFs were treated for 48 h with the indicated DNA-damaging agents. Induction of c-Raf phosphorylation by DNA damage leads to dephosphorylation of MEK1/2 and its substrate ERK1/2. *E*, regulation of rpS6 phosphorylation is S6K1/2-dependent. WT and S6K1/2^−/−^ MEFs were treated with the indicated DNA-damaging agents for 24 h. Cell extracts were analyzed by Western blot analysis with antibodies as shown. *F*, TSC2 is not required for the regulation of rpS6 in the presence of DNA damage. WT and TSC2^−/−^ MEFs were treated with the indicated DNA-damaging agents for 24 h. Cell extracts were analyzed by Western blot analysis with antibodies as shown. *High-exp.*, high exposure; *Low-exp.*, low exposure.

Akt activation in the presence of DNA damage has been reported to be independent of PI3K signaling but dependent on DNA-PK activation (18). Thus, we analyzed whether DNA-PK might transmit the DNA damage response to Akt to suppress rpS6. To examine the role of DNA-PK, we treated immortalized DNA-PK^−/−^ MEFs with DNA-damaging agents. Consistent with previous reports, DNA damage-induced Akt activation is diminished in DNA-PK^−/−^ MEFs (Fig. 4*A*) and, in contrast to control MEFs, DNA-PK^−/−^ MEFs are not able to down-regulate rpS6 phosphorylation in the presence of DNA damage. These findings indicate that DNA-PK activation is necessary to transmit the damage signal to Akt and regulate rpS6. Next, we analyzed signaling pathways downstream of Akt controlling rpS6 regulation in response to DNA damage. Initially, we focused on S6K1, which regulates protein synthesis and the cell cycle by phosphorylation of rpS6. It has been reported previously that S6K1 activity is also regulated by MAPK (20, 21) in addition to regulation by mTORC1 signaling. We found that inhibition of MEK signaling by a specific MEK inhibitor, AZD6244, reduced S6K1 kinase activity in NHDFs (Fig. 4*B*), suggesting that Akt might connect genotoxic stress to S6K1 by regulating MEK/MAP kinase activity in primary cells. It is well known that rapidly accelerated fibrosarcoma (Raf) family protein members (A-Raf, B-Raf, and c-Raf) are the main effectors recruited by GTP-bound Ras to activate the MEK-MAP kinase pathway (22). Studies have shown also that c-Raf function can be inhibited by Akt-dependent phosphorylation on serine 259 (23), which might lead to inhibition of MEK1/2, MAPK/ERK, and S6K1 signaling independently of the mTORC1 complex. To evaluate this signaling cascade under DNA-damaging conditions, cell extracts from the previous experiment using Akt1/2 DKO MEFs (Fig. 3*F*) were analyzed by Western blotting. As shown in Fig. 4*C*, in contrast to Akt1/2 DKO MEFs, the induction of c-Raf phosphorylation in wild-type cells by DNA damage leads to dephosphorylation of MEK1/2 and its substrate ERK1/2, indicating that the regulation of c-Raf by Akt plays an essential role in the cellular stress response in the absence of functional p53 (Fig. 4*C*). In addition to immortalized MEFs, we also found that, in primary NHDFs, DNA damage inhibited the c-Raf/MEK/MAPK/S6K1 signaling cascade (Fig. 4*D*), providing evidence that Akt might connect genotoxic stress to S6K1 by regulating c-Raf activity in primary cells. This signaling axis can therefore cooperate with the p53/mTORC1 axis in inactivating rpS6 in primary cells or provide a secondary mechanism for conserving energy in the absence of the p53-dependent regulation of this pathway.

MAPK has also been recognized as an activator of p90S6K, a serine/threonine kinase that regulates diverse cellular processes by phosphorylating a wide range of substrates, including rpS6 (24). To explore any possible redundancy between p90S6K and S6K1 for regulating rpS6 under conditions that damage DNA, we treated S6K1/2 DKO MEFs with various DNA-damaging chemotherapeutic drugs. We found that, in contrast to control MEFs, no phosphorylation of rpS6 is detectable in S6K1/2 DKO MEFs, indicating that S6K1/2 are the only enzymes that regulate phosphorylation of rpS6 in our experimental system (Fig. 4*E*). In addition, it has also been shown that activated MAPK can phosphorylate and inactivate TSC2 (25), raising the possibility that MAPK can also regulate S6K1 via a TSC1/2- and mTORC1- dependent pathway. To resolve this caveat, we compared p53^−/−^TSC^+/+^ to p53^−/−^TSC2^−/−^ MEFs in the presence of DNA damage. As shown in Fig. 4*F*, regardless of the TSC2 status of the MEFs, DNA damage suppressed rpS6 phosphorylation, supporting our model that, in response to DNA damage, cells can still restrict cellular metabolism by controlling rpS6 phosphorylation independently of TSC2, mTORC1, and p53.

##### Coordinate Functions of Both p53 and p63 Are Required for Controlling 4E-BP1 Dephosphorylation in Response to DNA Damage

**FIGURE 5.**
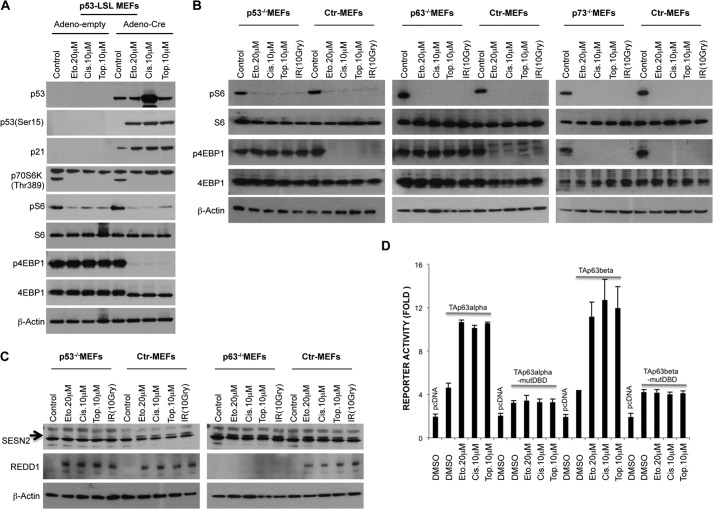
**p53^−/−^ and p63^−/−^ MEFs are not able to suppress 4E-BP1 phosphorylation in response to DNA damage.** *A*, re-expression of p53 restores DNA damage induced dephosphorylation of 4E-BP1. p53-lox-STOP-lox MEFs were infected with the indicated adenoviruses (*Adeno*). 24 h after infection, MEFs were treated with the indicated DNA-damaging agents for 24 h. Cell extracts were analyzed by Western blot analysis with antibodies as shown. *Eto*, etoposide; *Cis*, cisplatin; *Top*, topotecan. *B*, in contrast to p73^−/−^ MEFs, p53^−/−^ and p63^−/−^ MEFs are not able to inhibit 4E-BP1 phosphorylation in response to DNA damage. MEFs of each genotype were treated with the indicated DNA-damaging agents for 24 h. Cell extracts were analyzed by Western blot analysis with antibodies as shown. *IR*, ionizing radiation; *Ctr*, control. *C*, DNA damage-dependent Sestrin-2 induction is abolished in p53^−/−^ MEFs, and REDD1 induction is abolished in p63^−/−^ MEFs. MEFs of each genotype were treated with the indicated DNA-damaging agents for 24 h. Cell extracts were analyzed by Western blot analysis with antibodies as shown. *D*, luciferase reporter assay of the REDD1 promoter activity in NHDF. A REDD1 promoter-luciferase construct was cotransfected (Lipofectamine 2000) with the indicated plasmids. 24 h after transfection, cells were treated with topotecan (10 μm), etoposide (20 μm), cisplatin (10 μm), or dimethyl sulfoxide (DMSO) as a control for 24 h. Luciferase assay was performed according to the instructions of the manufacturer (Promega). *Error bars* show the mean ± S.D. for triplicate wells in a representative experiment. *mut*, mutant; *pcDNA*, cells transfected with empty plasmid.

Our data imply that a p53-dependent mechanism exists to control 4E-BP1 phosphorylation in the presence of DNA damage. We first tested whether p53 restoration might result in 4E-BP1 dephosphorylation in response to DNA damage. To restore p53 protein, we used a well established p53-lox-STOP-lox (LSL) MEF model (26). We infected p53-LSL MEFs with Adeno-empty as a control or Adeno-Cre for restoring p53 protein, and then MEFs were treated with DNA-damaging agents. As shown in Fig. 5*A*, restoring p53 resulted in dephosphorylation of 4E-BP1 only in the presence of DNA damage. Because overexpression of p53 alone did not result in hypophosphorylation of 4E-BP1, additional factors might be required for the complete regulation of 4E-BP1 phosphorylation. p53 belongs to the TP53 family, composed of the TP53, TP63, and TP73 genes, which exhibits strong structural homology and expression patterns. Importantly, a strong interplay has been described between p53 family members (27). For instance, all bind specifically to DNA response elements (p53RE), modulate gene expression, and, thus, determine cell fate outcome (28). Moreover, p53-mediated apoptosis is severely impaired in the absence of p63 and p73 in response to DNA damage (29). Thus, we explored the potential role of individual p53 family members in regulating mTORC1 signaling in the presence of DNA damage. MEFs deficient for one of the p53 family members and their control MEFs were treated with various DNA damage-inducing agents. As shown in Fig. 5*B*, in contrast to p73^−/−^ MEFs, p53^−/−^ and, surprisingly, p63^−/−^ MEFs were not able to suppress 4E-BP1 phosphorylation under DNA damage conditions. These findings indicate that, even in the presence of p53, p63 is necessary for the regulation of 4E-BP1 phosphorylation. This suggests that these genes might act together or in an obligatory parallel pathway to suppress 4E-BP1 phosphorylation subsequent to DNA damage. To understand genotoxic stress-induced 4E-BP1 inhibition more precisely in p53^−/−^ and p63^−/−^ MEFs, we first analyzed Sestrin expression, which was suggested to suppress mTORC1 signaling (10). Consistent with previous data, DNA damage-induced Sestrin2 expression is strongly abolished in p53^−/−^ but not p63^−/−^MEFs (Fig. 5*C*), indicating that induction of Sestrin2 alone is not adequate to suppress 4E-BP1 phosphorylation in the absence of p63. Another important stress-induced protein is REDD1, which was initially identified as a gene induced following stress stimuli that inhibit mTORC1 signaling (9, 30). Interestingly, previous data implicated a p53-independent up-regulation of REDD1 because TSC2^−/−^p53^−/−^ MEFs showed REDD1 induction upon stress stimuli (9, 31). Surprisingly, in contrast to p53^−/−^ and control MEFs, p63^−/−^ MEFs were not able to induce REDD1 expression (Fig. 5*C*), suggesting that, in response to DNA damage, induction of both Sestrin2 by p53 and REDD1 by p63 are necessary to inhibit 4E-BP1 phosphorylation.

##### ATM Kinase Drives REDD1 Induction by p63 in the Presence of DNA Damage

**FIGURE 6.**
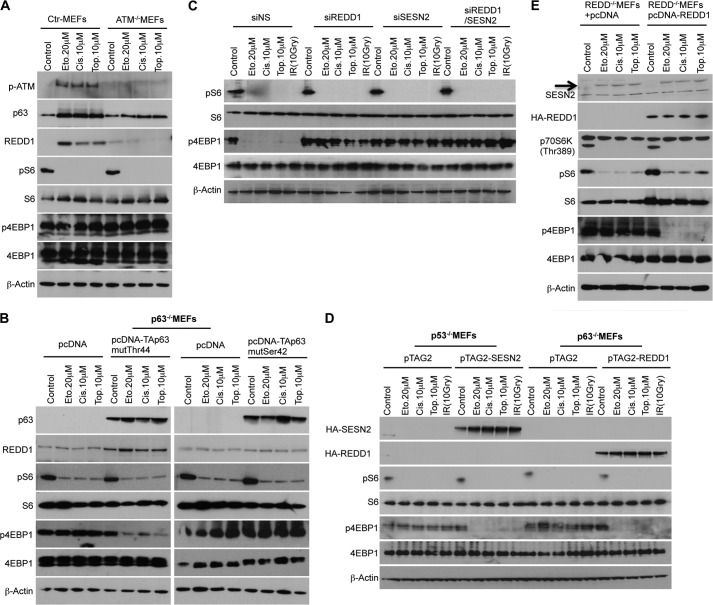
**ATM kinase drives REDD1 induction by p63 in response to DNA damage.** *A*, ATM^+/+^Arf^−/−^, and ATM^−/−^Arf^−/−^ MEFs were treated with the indicated DNA-damaging agents for 24 h. Cell extracts were analyzed by Western blot analysis with antibodies as shown. *Ctr*, control; *Eto*, etoposide; *Cis*, cisplatin; *Top*, topotecan. *B*, p63^−/−^ MEFs were electroporated with the indicated plasmids. 24 h after electroporation, p63^−/−^ MEFs were treated with the indicated DNA-damaging agents for 24 h. Cell extracts were analyzed by Western blot analysis with antibodies as shown. *C*, 48 h after single or combination knockdown of endogenous Sestrin-2 and REDD1, NHDFs were treated with the indicated DNA-damaging agents for 48 h. Cell extracts were analyzed by Western blot analysis with antibodies as shown. *IR*, ionizing radiation; *siNS*, non-silencing siRNA. *D*, MEFs of each genotype were electroporated with the indicated plasmids and incubated for 24 h. Subsequently, MEFs were treated with indicated DNA-damaging agents for 24 h. Cell extracts were analyzed by Western blot analysis with antibodies as shown. *E*, expression of REDD1 only suppresses phosphorylation of 4E-BP1 in the presence of DNA damage that induces Sestrin-2. Non-immortalized REDD1^−/−^ MEFs were electroporated with control (*pcDNA*) or pcDNA-REDD1 plasmids and treated with DNA-damaging agents as described above. Cell extracts were analyzed by Western blot analysis with antibodies as shown.

In response to DNA damage, the stress sensor protein kinase ATM is activated and phosphorylates many of its downstream targets, including p53 (32). Ultimately, this signaling cascade activates p53, leading to the transcriptional activation of target genes (*e.g.* Sestrin2) involved in cell cycle checkpoint activation, DNA damage repair, and/or apoptosis (33). Interestingly, we found a strong elevation of a REDD1 reporter when cotransfected with wild-type TAp63α in the presence of DNA damage but not after cotransfection with plasmids having mutations in the DNA binding domain (Fig. 5*D*). Consistent with the idea that REDD1 is regulated downstream of ATM, DNA damage failed to induce REDD1 in ATM-deficient cells (Fig. 6*A*). These data suggest that the posttranslational modification of p63 by ATM might contribute to increased REDD1 induction. We identified two putative ATM phosphorylation sites at Ser-42 (SQ) and Thr-44 (TQ) in the N terminus of p63. By mutational analysis of these putative ATM phosphorylation sites on p63, we found that Ser-42 is the essential site for the induction of REDD1 by DNA damage (Fig. 6*B*). In contrast to the p63 construct mutated at the Thr-44 site (T44A), the introduction of the S42A p63 plasmid in p63^−/−^ MEFs did not induce REDD1 reporter activity under DNA damage conditions (data not shown). This result supports our initial finding that, upon DNA damage, ATM most likely phosphorylates p63 at Ser-42 and, in turn, that phosphorylated p63 induces REDD1 expression.

##### Sestrin2 and REDD1 Induction by DNA Damage Are Required for Controlling 4E-BP1 Phosphorylation

To show a possible link between Sestrin-2 induction by p53 and REDD1 induction by p63 directly, we knocked down the Sestrin-2 and REDD1 genes alone or in combination in NHDFs (data not shown). As shown in Fig. 6*C*, in contrast to control cells, single knockdown of Sestrin2 or REDD1, as well as combined knockdown of both genes, abrogated the regulation of 4E-BP1 phosphorylation following DNA damage, thus providing direct evidence that cells indeed need Sestrin-2 as well as REDD1 expression to suppress 4E-BP1 phosphorylation in response to DNA damage. Moreover, overexpression of Sestrin2 in p53^−/−^ MEFs and REDD1 in p63^−/−^ MEFs suppressed 4E-BP1 phosphorylation only under DNA damage conditions. This supports our finding that the induction of both proteins by DNA damage is necessary for 4E-BP1 regulation (Fig. 6*D*). In addition, introduction of REDD1 into non-immortalized REDD1^−/−^ MEFs suppressed 4E-BP1 phosphorylation only under conditions of DNA damage, which induce Sestrin2 through the p53-dependent parallel pathway (Fig. 6*E*). These data provide additional evidence that the induction of both proteins at the same time is essential to control 4E-BP1 phosphorylation.

##### Redd1 and Sestrin2 Ensure Proper Control of mTORC1 Signaling in Response to DNA Damage

**FIGURE 7.**
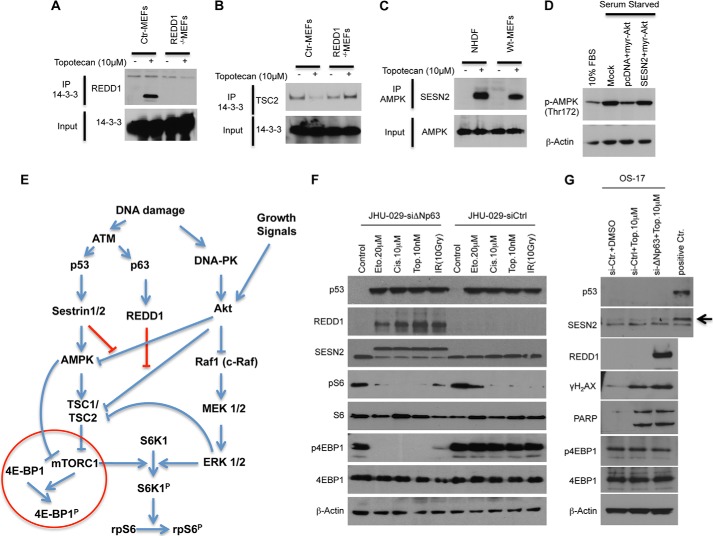
**Induction of Sestrin-2 by p53 and REDD1 by p63 prevent AKT from inhibiting AMPK and the TSC1/2 complex.** *A*, the interaction of endogenous REDD1 and 14-3-3 is DNA damage-dependent. WT and REDD1^−/−^MEFs were treated with topotecan (10 μm) for 24 h, followed by immunoprecipitation (*IP*) for endogenous 14-3-3. Extracts were analyzed by Western blot analysis for REDD1 and 14-3-3 proteins as shown. *Ctr*, control. *B*, induction of REDD1 is essential to abrogate the association of TSC2/1 4-3-3 proteins following DNA damage. WT and REDD1^−/−^ MEFs growing in 10% serum were treated with topotecan (10 μm) for 24 h followed by immunoprecipitation for endogenous 14-3-3. Extracts were analyzed by Western blot analysis with TSC2 antibody as shown. *C*, in the presence of DNA damage, Sestrin-2 binds AMPK. NHDFs and MEFs were treated with topotecan (10 μm) for 24 h, followed by immunoprecipitation for endogenous AMPK. Extracts were analyzed by Western blot analysis. *D*, Sestrin-2 expression rescues the inhibition of AMPK phosphorylation by myr-AKT. NHDFs were cotransfected with myr-Akt along with pcDNA or Sestrin-2 expression vectors and serum-starved for 24 h. Cell extracts were analyzed by Western blot analysis with antibodies as shown. *E*, schematic illustrating the complex regulation of mTORC1 signaling by DNA damage. *F* and *G*, multiple mechanisms in tumors that suppress p53/p63 responses also abrogate the ability of the cancer cell to control mTORC1 in response to DNA damage. *F*, JHU- 029 cells were transfected with the indicated siRNAs (Dharmacon). 72 h after transfection, cells were treated for 24 h with the indicated DNA-damaging agents. Cell extracts were analyzed by Western blot analysis with antibodies as shown. *G*, OS-17 were transfected with the indicated siRNAs (Dharmacon). 72 h after transfection, cells were treated for 24 h with the indicated DNA-damaging agents. Cell lysates from topotecan-treated NHDFs was used as a positive loading control (*positive Ctr.*). Cell extracts were analyzed by Western blot analysis with antibodies as shown.

We observed that DNA damage, regardless of p53 status, activates DNA-PK, which results in the inhibition of rpS6 phosphorylation via an Akt/c-Raf/MEK/MAPK(Erk)/S6K1-dependent, mTORC1-independent pathway. Under physiologic conditions or experimental settings, cells are faced with growth stimuli while extrinsic or intrinsic cellular stress or damage exists. The positive regulation of mTORC1 by growth factor-induced TSC2 inhibition occurs primarily through the activation of the PI3K/Akt pathway, whereby Akt directly phosphorylates and inhibits TSC2 (34, 35). TSC phosphorylation by Akt promotes TSC2/14-3-3 association and, thereby, inhibits TSC1/2 function (31). For instance, in response to hypoxia, induced REDD1 binds 14-3-3, resulting in TSC2/14-3-3 dissociation, TSC1/2 activation, and mTORC1 inhibition (31). Thus, REDD1 can indirectly inhibit mTORC1 even in the presence of constitutive Akt activation. To test whether REDD1 also binds 14-3-3 under classical DNA damage conditions, we treated control as well as REDD^−/−^ MEFs with the DNA-damaging cancer chemotherapeutic agent topotecan. Coimmunoprecipitation of 14-3-3 with damage-induced endogenous REDD1 implies that, as under hypoxic conditions, REDD1 blocks the positive signal of Akt to regulate mTORC1 by sequestering 14-3-3, leading to the reactivation of TSC function (Fig. 7*A*). Furthermore, we confirmed that endogenous REDD1 is required for DNA damage-induced TSC2/14-3-3 dissociation (Fig. 7*B*). It has also been shown that AMPK, negatively regulated by Akt (36, 37), can inhibit mTORC1 via a TSC1/2-independent mechanism. Along with others, we found that DNA damage-induced Sestrin-2 interacts with AMPK (10) (Fig. 7*C*). This interaction between Sestrin2 and AMPK might lead to conformational changes that could prevent Akt from inhibiting the kinase activity of AMPK. To test this, NHDFs were cotransfected with myr-Akt along with the pcDNA or Sesn2 expression vectors. As shown in Fig. 7*D*, myr-Akt expression significantly reduced the activating phosphorylation on AMPK (Thr-172), and coexpression of Sestrin2 with myr-Akt rescued the myr-Akt-induced dephosphorylation of AMPK. Together, our results indicate that the regulation of 4E-BP1 phosphorylation requires both p53 and p63 in response to DNA damage. The induction of Sestrin2 by p53, and of REDD1 by p63, prevents Akt from inhibiting AMPK and TSC1/2, ensuring proper control of mTORC1 signaling in response to DNA damage even in the presence of growth stimuli (Fig. 7*E*).

## DISCUSSION

In this study, we demonstrate a novel role of Akt in the regulation of mTORC1 signaling in the presence of DNA damage, whereas AMPK and p53 are not potent inhibitors of mTORC1 signaling toward S6K1 but, rather, only make a partial contribution by regulating 4E-BP1 phosphorylation. Several recent studies have implicated Akt in modulating DNA damage responses and genome stability and suggest that Akt can be activated in response to DNA damage through the action of the PI3K-like kinases ATM, ataxia telangiectasia and Rad3-related protein (ATR), and DNA-PK. Conversely, active Akt can promote DNA repair via non-homologous end joining and inhibit checkpoint signaling and repair via recombination through multiple mechanisms and targets (19). Interestingly, the physiological requirement of Akt activation and, in turn, regulation of mTORC1 signaling has not been questioned under conditions of DNA damage. Here, we describe that the Akt pathway plays a key role in the regulation of rpS6 phosphorylation and is essential in p53-deficient cells in response to DNA damage. By comparing several immortalized knockout MEFs, we discovered that the phosphorylation of ribosomal protein S6 is mediated by a DNA-PK/Akt/cRaf/MEK/MAPK/S6K1 signaling cascade in the presence of DNA damage.

It should be noted that, under physiologic conditions or experimental conditions, cells are faced with growth stimuli while extrinsic or intrinsic cellular stress or damage exists. We found that DNA damage increased Akt kinase activity, as measured by increased Akt phosphorylation. Growth factors also increase Akt kinase activity, whereby Akt activates mTORC1 signaling by inhibition of the AMPK and TSC complexes. Thus, cells must overcome this paradox in the presence of DNA damage. Therefore, we hypothesized that there may be a direct relationship between Sestrin and REDD1 induction and, in turn, Akt kinase in the presence of DNA damage. Indeed, we demonstrate the ability of DNA damage-induced REDD1 to disrupt TSC2/14-3-3 binding, therefore, provides a means to rapidly extinguish mTORC1 activation even in the presence of growth factors that activate Akt. On the other hand, on the basis of our results, we speculate that the interaction between Sestrin2 and AMPK might lead to conformational changes that could also prevent Akt from inhibiting the kinase activity of AMPK. Thus, via disabling the PI3K/Akt/AMPK/TSC axis and activating the DNA-PK/Akt/c-Raf/MEK/MAPK signaling cascade, cells ensure proper control of mTORC1 signaling in the presence of DNA damage under conditions where Akt activation is maintained by exogenous growth factors.

We further demonstrate another level of dysregulation of TORC1 in tumors that are p53 wild-type. Specifically, many cancers overexpress the oncogenic ΔNp63, a dominant negative isoform of TAp63. JHU-029 squamous carcinoma cells overexpressing ΔNp63 fail to negatively regulate TORC1 signaling under conditions of DNA damage. In these cells, the down-regulation of ΔNp63 resulted in up-regulation of p53/Sestrin 2 as well as p63/REDD1 only in cells where DNA damage was induced (Fig. 7*F*). In contrast, in OS-17 cells that are unable to induce p53 under conditions of DNA damage, the down-regulation of ΔNp63 resulted in a robust REDD1 induction but no inhibition of 4EBP phosphorylation (Fig. 7*G*). These findings support the dual roles of p53/p63 in mediating TORC1 inhibition following DNA damage and suggest that multiple mechanisms in tumors that suppress p53/p63 responses also abrogate the ability of the cancer cell to control TORC1, and, hence, cap-dependent translation, under conditions of DNA damage.

Thus, in cells with wild-type p53, TORC1 signaling is presumably suppressed as both 4EBP and S6K pathways are inhibited, and translation will be largely suppressed. Because TORC1 signaling regulates cyclin-dependent kinase (CDK)/cyclins (38) and p53 positively regulates the cyclin/CDK inhibitor p21, these processes coordinate cell cycle arrest and energy conservation under conditions of DNA damage. The situation is similar to that induced by TOR kinase inhibitors (39–41) where both the 4EBP and S6K pathways are inhibited equally but with one difference. DNA damage induces the activation of Akt, whereas TOR kinase inhibitors repress the full activation of Akt by inhibiting TORC2 activity (39, 40). In contrast, the effect of DNA damage in p53 mutant lines is more reminiscent of the effects rapamycin has on TORC1 signaling. In many cell lines, rapamycin causes complete abrogation of the pS6 signal but has relatively little effect on decreasing p4EBP. Also, in many cell lines, rapamycin induces the hyperphosphorylation of Akt (Ser-473). However, rapamycin causes only a relatively small decrease in global translation (∼20%) that is associated with a transient increase in G_1_ phase cells and poor suppression of cell proliferation. The biological consequences of the unequal suppression of these two pathways downstream of TORC1 have not been addressed. In addition to rpS6, S6Ks phosphorylate and activate eIF4B, required for efficient recruitment of ribosomes to mRNA (5). Activated eIF4B stimulates both the ATPase and RNA helicase activities of eIF4A (5) and elongation factor 2 (eEF2). eIF4B is required for ribosome binding on an mRNA-containing secondary structure, and down-regulation of eIF4B results in the selective inhibition of translation of mRNAs having complex structures associated with their 5′ UTR (5, 42). Thus, the consequences of DNA damage in the context of wild-type p53 or mutant p53 may be very different. Most likely, in p53 mutant tumors, maintained proliferation, mediated by maintained translation, would lead to further genome plasticity. Importantly, drugs (*e.g.* Akt inhibitors) that specifically inhibit enzymes in the TORC1-independent pathway that regulates S6 phosphorylation in the presence of DNA damage are being combined with conventional cytotoxic DNA-damaging agents. Potentially, in the context of cells with inactivated p53, such combinations could abrogate DNA damage-induced signaling to suppress S6K activity, maintain translation, and impact cellular responses to therapy. The effect of maintaining inactive 4E-BP and active S6 on cellular responses to DNA-damaging agents warrants further investigation. Furthermore the lack of phospho-S6, or changes in S6 phosphorylation, following drug treatments, is not necessarily indicative of mTORC1 signaling. Thus, dephosphorylation of 4E-BP1 should be considered as the more appropriate biomarker for mTORC1 activity.

## Footnotes

2 The abbreviations used are:

mTOR: mammalian target of rapamycin
mTORC: mammalian target of rapamycin complex
TSC: tuberous sclerosis complex
MEF: mouse embryonic fibroblast
NHDF: normal human dermal fibroblast
DKO: double knockout
ATM: ataxia telangiectasia mutated
AMPK: AMP-activated protein kinase
LSL: lox-STOP-lox.
